# Spatial prediction and drivers of surface soil organic carbon density in subtropical montane forests

**DOI:** 10.7717/peerj.21704

**Published:** 2026-09-07

**Authors:** Zhongchi He, Yiting He, Xiaorong Chen, Yanyun Xiong, Siyu Wang, Yihong Liu, Jiasen Wu, Xinli Chen, Youchao Chen, Yanjiang Cai

**Affiliations:** 1 State Key Laboratory for Development and Utilization of Forest Food Resources, Zhejiang A&F University, Hangzhou, China; 2 College of Environment and Resources, College of Carbon Neutral, Zhejiang A&F University, Hangzhou, China; 3 Qianjiangyuan-Baishanzu National Park Qingyuan Conservation Center, Lishui, China

**Keywords:** Soil organic carbon density, Machine learning, Structural equation modelling, Subtropical montane forest, Carbon stock mapping

## Abstract

Soil organic carbon (SOC) represents the largest terrestrial carbon (C) pool, and even small changes in its stock can exert great influences on regional and global C cycles. Quantifying the spatial distribution of forest SOC and identifying its key drivers are therefore central to climate change science. In this study, we collected 150 topsoil samples (0–20 cm) across six vegetation types spanning an elevation gradient of 500–1,900 m in Baishanzu National Park, Zhejiang Province. Soil organic carbon density (SOCD) was determined alongside litter stock, and the activities of three cellulolytic hydrolases—α-glucosidase (AG), β-glucosidase (BG), and cellobiohydrolase (CBH). Ten environmental covariates derived from remote sensing, climate, and topographic data were used to predict SOCD. Three machine learning models—random forest (RF), boosted regression trees (BRT), and eXtreme Gradient Boosting (XGBoost)—were evaluated using repeated nested five-fold cross-validation and nested nearest neighbor distance matching cross-validation (NNDM-CV), with hyperparameter tuning restricted to the corresponding outer-training partitions. The three algorithms showed broadly comparable predictive performance across the two validation schemes. Based on the XGBoost model, the predicted mean surface SOCD across the park was 108.6 t C hm^−2^, with pixel-level values ranging from 39.4 to 160.6 t C hm^−2^. The total surface SOC stock was approximately 5. 4 × 10^6^ t C. The bootstrap ensemble indicated moderate resampling-based model uncertainty, with a mean pixel-level standard deviation of 10.0 t C hm^−2^ and a mean coefficient of variation of 9.5%. SHAP analysis showed that vegetation-related variables dominated SOCD prediction. Structural equation modelling showed that the vegetation index had both a positive direct effect on SOCD and an indirect positive effect mediated by litter stock, whereas the C-acquiring enzyme index was negatively associated with SOCD. These findings improve our understanding of the spatial distribution of SOCD in subtropical montane forests and provide a scientific basis for regional C stock assessment and forest ecosystem management.

## Introduction

Since the Industrial Revolution, human activities—particularly the extensive use of fossil fuels—have led to a significant increase in atmospheric CO_2_ concentrations (Calvin et al., 2023). Mitigating climate change by reducing anthropogenic CO_2_ emission sources and enhancing carbon (C) sinks in natural ecosystems has become a widespread global consensus. The soil organic carbon (SOC) pool stores approximately two to three times the amount of C in the atmosphere; even minor changes in the SOC pool can exert great effects on the global climate (Jobbágy & Jackson, 2000). Therefore, accurately estimating SOC stocks and their spatial distribution is of critical importance for understanding global C cycling processes and assessing the C sequestration potential of ecosystems. Forests play a major role in global C sequestration, and forest soils constitute an important C pool within the global forest C budget (Zhang et al., 2024). Their substantial C sequestration capacity is widely recognized as one of the most cost-effective, safe, and efficient means of C fixation (Griscom et al., 2017). Consequently, elucidating forest SOC stocks and their controlling factors is of great significance for estimating regional C budgets and for formulating forest management strategies.

However, research on the spatial distribution of forest SOC stocks still faces considerable challenges. Traditional approaches—such as soil type–based methods, biogeochemical models, and simple regression-based statistical models—can be constrained by coarse mapping units and limited representation of nonlinear covariate–SOC relationships at regional scales (Zhu et al., 2025); geostatistical methods, by contrast, are well suited to change-of-support and to quantifying prediction uncertainty (*e.g*., kriging variance and prediction intervals) (Szatmári, Pásztor & Heuvelink, 2021; Wadoux & Heuvelink, 2023). In recent years, advances in remote sensing technologies and machine learning algorithms have provided new tools for SOC stock estimation and digital soil mapping at regional to national scales (Xia, McSweeney & Wander, 2022; Radočaj, Gašparović & Jurišić, 2024). However, the predictive performance and applicability of different models still vary substantially in mountainous regions characterized by complex terrain and high vegetation heterogeneity. Among the various machine learning algorithms, Random Forest (RF), Boosted Regression Tree (BRT), and eXtreme Gradient Boosting (XGBoost) are capable of handling nonlinear relationships and variable interactions and have been widely applied in ecological studies (Yang et al., 2016; Emadi et al., 2020; Chen, Wang & Zhu, 2024). In particular, XGBoost is an ensemble model based on Gradient Boosted Decision Trees (GBDT) that incorporates regularization techniques to enhance prediction accuracy and mitigate overfitting under complex, multi-factor-driven conditions (Chen & Guestrin, 2016). Recent SOC mapping studies have shown that XGBoost has strong or competitive predictive performance relative to RF and BRT in cropland and grassland systems (Emadi et al., 2020; Geng et al., 2024). Nevertheless, relatively few studies have evaluated these models in montane forest ecosystems where steep environmental gradients and canopy structural complexity pose additional challenges.

Beyond prediction accuracy, understanding the ecological mechanisms governing SOC spatial variation is equally important. In forest ecosystems, surface SOC is regulated by aboveground litter inputs, belowground root C inputs, and microbial decomposition (Prescott & Grayston, 2013; Cotrufo & Lavallee, 2022; Liu et al., 2025). Despite their strong predictive power, machine learning models are primarily predictive tools. Model-agnostic interpretation methods such as SHapley Additive exPlanations (SHAP) can rank the contribution of each covariate to the predicted spatial pattern, but these importance measures cannot separate direct from indirect effects among correlated drivers. Structural equation modelling (SEM) is complementary to this predictive interpretation. Rather than explaining the machine-learning map, SEM tests a hypothesized ecological network by quantifying the direct and indirect pathways through which drivers act on SOC simultaneously, and has been applied widely to SOC studies at the plot and landscape scale (Satdichanh et al., 2023; Li et al., 2024). The two methods therefore serve distinct purposes. SHAP identifies which covariates drive the spatial prediction, whereas SEM evaluates how selected process variables are linked to SOC through measured ecological processes. However, studies that couple high-resolution spatial prediction from machine learning with mechanistic pathway analysis *via* SEM remain scarce, particularly in the subtropical montane region of China.

Qianjiangyuan-Baishanzu National Park is a key ecological functional area in the Yangtze River Delta region, and provides critical ecological services including water conservation, biodiversity maintenance, forest C sequestration, and climate regulation (Shen, Li & Ma, 2021). The Baishanzu section of the park is situated within the Wuyi Mountains Biodiversity Priority Conservation Area of China, where mountainous terrain accounts for over 90% of the total area. Despite the park’s ecological significance, previous research conducted in the Baishanzu section has primarily focused on biodiversity conservation (She et al., 2022), protection of endemic species (Liu & Liu, 2021), and the altitudinal zonation of forest vegetation (Wei et al., 2011); a mechanistic assessment of its soil C stock and spatial drivers has not been conducted.

Accordingly, the present study conducted a soil survey in Baishanzu National Park from July to November 2024, establishing 150 sampling sites from which topsoil samples (0–20 cm depth) were collected to determine SOCD. The main objectives were: (1) to benchmark RF, BRT, and XGBoost for SOCD spatial prediction in this complex montane area and produce a park-wide SOCD map using the selected model; (2) to characterize the spatial distribution of SOCD across vegetation types; and (3) to identify the main drivers of SOCD distribution and explore the underlying mechanism using SEM.

## Materials and Methods

### Study area

Baishanzu National Park (119°07′45′′–119°19′20′′ E, 27°37′45′′–27°50′30′′ N) is located in southern Zhejiang Province, China, with a total area of 505 km^2^. The terrain is predominantly montane, with elevations ranging from 291 to 1,929 m a.s.l. The park lies within the mid-subtropical monsoon climate zone, characterized by distinct seasons, abundant precipitation (mean annual precipitation approximately 2,341 mm), and a mean annual temperature of 12.8 °C. Extreme temperatures range from −13.6 to +30.5 °C. Annual frost persists for approximately 55 days (November to March). Soils are predominantly red soils (Ultisols/Oxisols) below 800 m and yellow soils (Inceptisols) above 800 m. The vegetation follows a well-developed altitudinal zonation: evergreen broadleaf forest and warm-temperate coniferous forest below ~1,000 m; mixed evergreen–deciduous broadleaf and temperate coniferous forest between 1,000 and 1,500 m; and montane shrub-grassland above 1,500 m.

### Soil sampling and laboratory analysis

**Figure 1:**
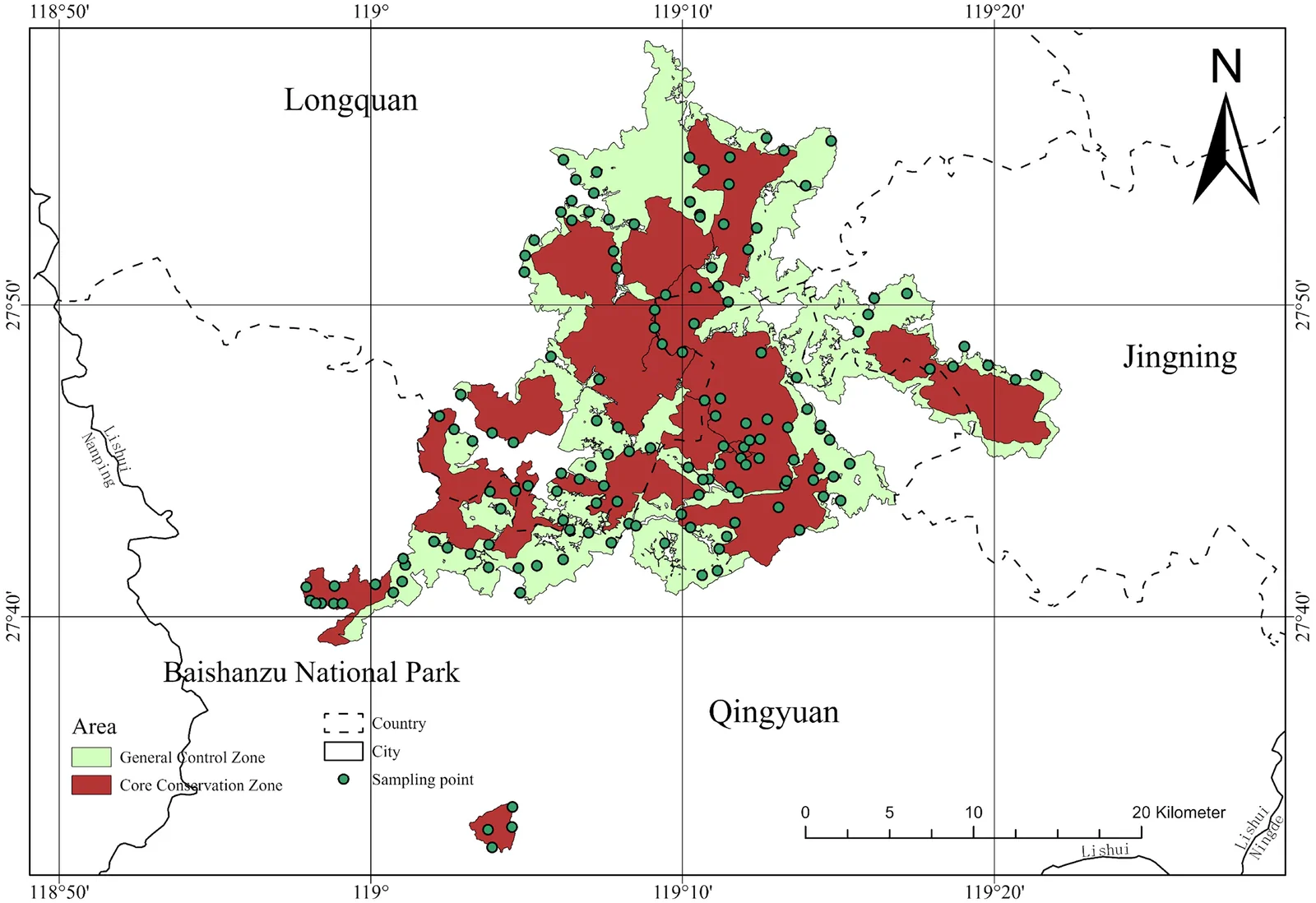
Location of Baishanzu National Park and distribution of the 150 soil sampling sites.

During July–November 2024, 150 sampling sites were established across the whole study region (Fig. 1). Field sampling was conducted with the authorization and support of Qianjiangyuan-Baishanzu National Park Qingyuan Conservation Center. The sampling design was stratified and representative rather than strictly probability-based. Sites were selected to cover the six major vegetation types and the main elevation gradient from 500 to 1,900 m, while also considering field accessibility, sampling safety, and protected-area restrictions. Some steep, remote, or strictly protected areas were not sampled because intensive field sampling was not feasible or permitted. A minimum inter-site spacing of 1 km was maintained where possible. At each site, basic metadata were recorded, including geographic coordinates (GPS), elevation, slope angle and aspect, slope position, dominant plant species by canopy layer, vegetation cover, and bare-rock fraction. At each of the sampling sites, all aboveground litter (including freshly fallen leaves, twigs, bark fragments, and partially decomposed organic material) was collected from a 1 m × 1 m sub-quadrat within the 20 m × 20 m plot. Another three 1 m × 1 m sub-quadrats were then placed to represent general plot conditions while avoiding atypical microsites such as tree stems, exposed rocks, or animal disturbance. In each selected 1 m × 1 m sub-quadrat, surface litter was gently removed, and one soil sample was collected from the 0–20 cm mineral soil layer using a soil auger. Disturbed soil samples collected from the three sub-quadrats were homogenized into one representative composite sample for each site. Separately, in each selected 1 m × 1 m sub-quadrat, one undisturbed soil core was collected from the 0–20 cm soil layer using a cutting ring for bulk density (BD) determination. The composite disturbed soil sample was passed through a 2 mm sieve to remove plant debris and gravel, and then divided into two portions. One portion was stored at 4 °C for enzyme activity assays, whereas the other was air-dried at room temperature and stored in zip-lock bags for subsequent analyses of SOC and soil pH. SOC content was determined using an elemental analyzer (VarioMAX C/N; Elementar, Frankfurt, Germany). SOCD (t C hm^−2^) for the 0–20 cm layer was calculated as:

$\rm SOCD = SOC\times BD\times D$

where SOC is organic C content (%), BD is bulk density (g cm^−3^), and D is the soil thickness (cm).

Litter samples were oven-dried at 65 °C to constant mass (approximately 48 h) and weighed to calculate litter stock (g m^−2^). Soil pH was measured in a 1:2.5 (w/v) soil-to-water suspension using a pH meter (Mettler Toledo, Columbus, Ohio, USA). Three cellulolytic hydrolase activities were assayed fluorometrically using methylumbelliferyl (MUB)-linked substrates following the protocol of Bell et al. (2013). α-Glucosidase (AG) was assayed with 4-MUB-α-d-glucopyranoside, β-glucosidase (BG) with 4-MUB-β-d-glucopyranoside, and cellobiohydrolase (CBH) with 4-MUB-β-d-cellobioside.

### Remote-sensing and environmental covariates

**Figure 2:**
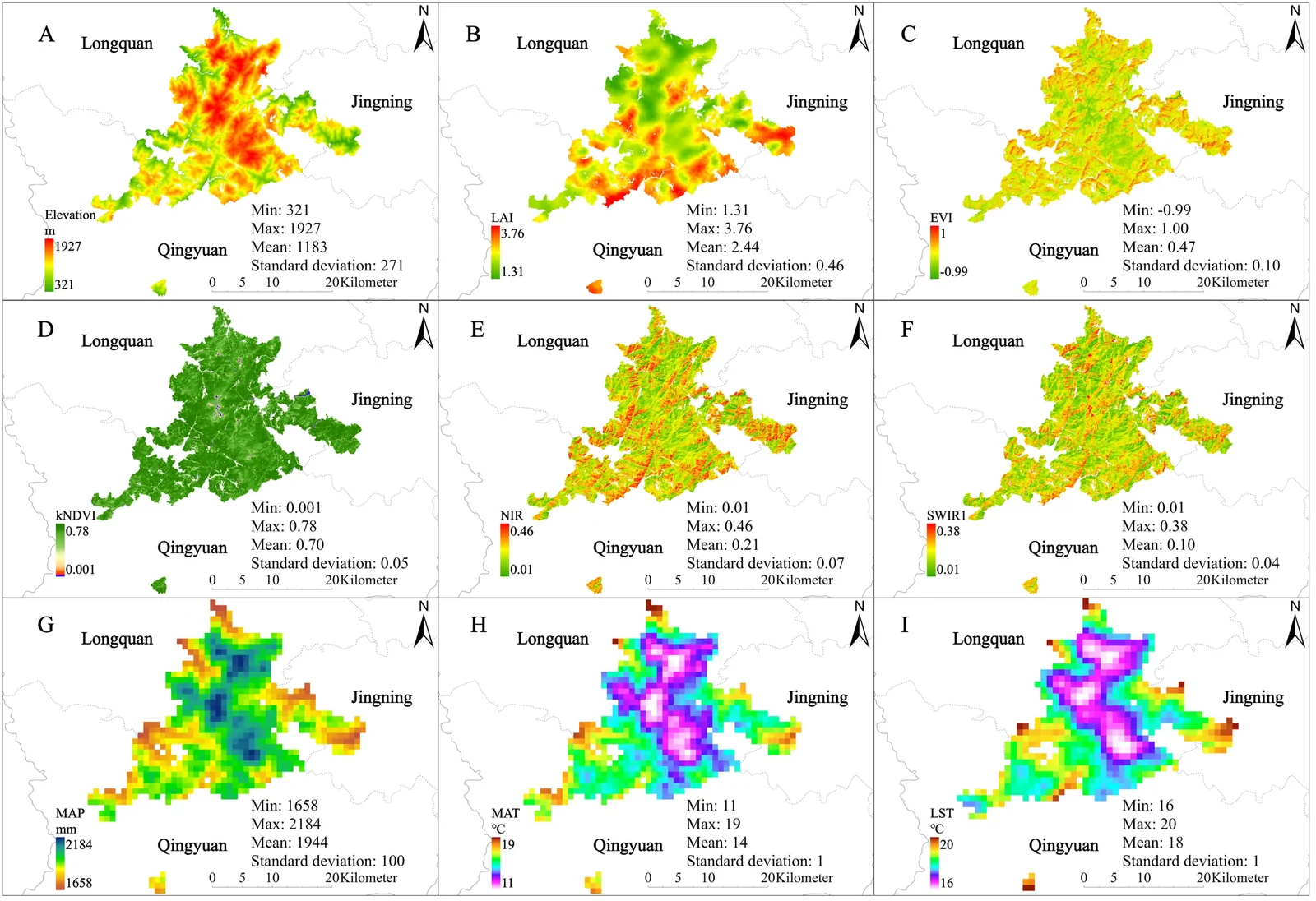
Maps of the environmental factors. (A) Elevation; (B) LAI, leaf area index; (C) EVI, enhanced vegetation index; (D) kNDVI, kernel normalised difference vegetation index; (E) NIR, near-infrared; (F) SWIR1, shortwave infrared 1; (G) MAP, mean annual precipitation; (H) MAT, mean annual temperature; (I) LST, land surface temperature.

Following Meng et al. (2024), ten environmental predictors were assembled from multi-source satellite and ancillary datasets. Specifically, we chose leaf area index (LAI), enhanced vegetation index (EVI), kernel normalized difference vegetation index (kNDVI), near-infrared (NIR), shortwave infrared 1 (SWIR1) bands, mean annual precipitation (MAP), mean annual temperature (MAT), land surface temperature (LST), elevation derived from digital elevation model (DEM), and vegetation type derived from the vector dataset of the Second National Land Survey of Lishui City (note: as this dataset is classified, the raw data of vegetation type are not presented in this study). All predictors were selected on the basis of established theoretical links to SOC storage (Meng et al., 2024). All remote-sensing data were acquired and processed on the Google Earth Engine (GEE) cloud platform using multi-year composites (2017–2023) to reduce phenological noise. Sentinel-2 Surface Reflectance imagery (COPERNICUS/S2_SR) and Landsat 8/9 Collection 2 Level-2 imagery were filtered for cloud cover <10%, radiometrically corrected, and composited as annual median images. Vegetation type data were converted and reclassified to match the spatial extent and resolution of the remote-sensing covariates. ERA5-Land climate variables were resampled to 30 m to match the remote-sensing spatial resolution. All layers were clipped to the park boundary, reprojected to WGS 1984 geographic coordinate system, and exported as GeoTIFF (Fig. 2).

### Machine learning models

Three ensemble machine learning models were employed for SOCD spatial prediction, including random forest (RF), boosted regression trees (BRT), and XGBoost. The same set of environmental covariates was used for all models. For machine-learning model fitting, continuous predictor variables were retained in their original units because RF, BRT, and XGBoost are tree-based methods and are insensitive to predictor scaling (Hastie, Tibshirani & Friedman, 2009). Vegetation type was treated as a categorical variable and encoded as dummy variables. To ensure a fair comparison among algorithms and to keep hyperparameter tuning separate from performance evaluation, we adopted a nested cross-validation scheme. In the outer loop, model performance was evaluated using both repeated five-fold cross-validation and nearest neighbor distance matching cross-validation (NNDM-CV) (Milà et al., 2022). Within each outer training partition, key hyperparameters were tuned in a separate inner five-fold cross-validation over pre-defined parameter grids, and the combination with the lowest inner cross-validated root mean squared error was selected; the model was then refitted on that outer training partition with the selected hyperparameters and evaluated on the held-out outer observations, which were never seen during tuning. All 150 observations were used for model evaluation through this nested procedure. Model accuracy was assessed using the Nash-Sutcliffe efficiency form of R^2^, root mean squared error (RMSE, t C hm^−2^), and mean absolute error (MAE, t C hm^−2^), calculated from observed and predicted SOCD values:

${R^2} = 1 - \left[ {\mathop \sum \limits_{i = 1}^n {{\left( {{y_i} - {{\hat y}_i}} \right)}^2}/\mathop \sum \limits_{i = 1}^n {{\left( {{y_i} - \bar y} \right)}^2}} \right]$

$RMSE = \sqrt {\left[ {\displaystyle{{\mathop \sum \nolimits_{i = 1}^n {{\left( {{y_i} - {{\hat y}_i}} \right)}^2}} \over n}} \right]}$

$$MAE = \displaystyle{1 \over n}\mathop \sum \limits_{i = 1}^n \left| {{y_i} - {{\hat y}_i}} \right|$$

where $\rm y_i$ and $ {\hat {\rm y}}_{i}$ denote the observed and predicted SOCD values for sample i, respectively, $\overline{\rm y}$ is the mean of the observed SOCD values, and *n* is the number of observations.

The selected XGBoost model was then refitted using all 150 observations and applied to wall-to-wall raster covariates to generate the final SOCD prediction map for the study area. Bootstrap-derived model uncertainty was assessed using a 100-member XGBoost ensemble. For each pixel, the mean prediction, standard deviation, and coefficient of variation were calculated from the ensemble predictions. These dispersion statistics represent bootstrap-derived prediction variability and were not interpreted as calibrated prediction intervals or total predictive uncertainty. Total SOC stock was calculated by multiplying pixel-level SOCD by the corresponding pixel area and summing across all valid pixels within the study boundary.

### Statistical analysis

Statistical analysis and data preprocessing were conducted in R 4.4.3 (R Core Team, 2025). Machine learning models were implemented using the *xgboost*, *randomForest*, and *gbm* packages. SHAP values were calculated to quantify the contribution of each predictor to SOCD prediction. To further explore the potential pathways through which environmental covariates were associated with SOCD, a structural equation model (SEM) was constructed using the full dataset of 150 plots. The conceptual model was developed based on the ecological hypothesis that vegetation condition may influence SOCD directly and indirectly through litter input and C-acquiring enzyme activity. Based on the SHAP results, the main vegetation-related predictors, including EVI, NIR, LAI, and kNDVI, were summarized by principal component analysis (PCA), and PC1 was used as a vegetation-index composite in the SEM. The SHAP results were used only to guide the choice of vegetation predictors; the SEM was fitted independently of the prediction model, as a separate test of the hypothesized association structure among field-measured variables. Litter stock was included as an indicator of organic matter input, and the C-acquiring enzyme index was specified as a latent variable indicated by AG, BG, and CBH activities. All variables were z-score standardized before model fitting. The SEM was fitted by maximum likelihood estimation using the *lavaan* package in R, and model fit was evaluated using the chi-square test, CFI, RMSEA, and SRMR. Spatial analysis and mapping were performed in ArcGIS Pro 3.2. All spatial outputs are referenced to the WGS 1984 geographic coordinate system.

## Results

### Descriptive statistics of soil and litter sample properties

Overall, the mean SOC content of soil samples was 5.7 ± 2.8%, with BD averaging 0.80 ± 0.24 g cm^−3^ and soil pH averaging 4.9 ± 0.4. Mean litter stock across all sites was 620 ± 280 g m^−2^ (Table 1). The properties of soil and litter samples varied across the six vegetation types sampled in Baishanzu National Park. In general, shrubland had the highest SOC content while bamboo forest had the lowest; soil pH was highest in bamboo forest and lowest in coniferous forest; BD was highest in bamboo forest and lowest in shrubland; litter stock was greatest in broadleaf forest and lowest in bamboo forest; for all three enzyme activities, bamboo forest showed the highest values whereas broadleaf forest showed the lowest, a pattern consistent across AG, BG, and CBH (Table 1).

**Table 1:** Properties of soil and litter samples across vegetation types in Baishanzu National Park.

Vegetation type	SOC (%)	pH	BD (g cm^–3^)	Litter stock (g m^–2^)	AG (nmol g^–1^ h^–1^)	BG (nmol g^–1^ h^–1^)	CBH (nmol g^–1^ h^–1^)
Coniferous forest (*n* = 43)	5.7 ± 2.5	4.6 ± 0.4	0.83 ± 0.19	610 ± 240	23 ± 12	60 ± 31	11.9 ± 5.1
Mixed forest (*n* = 34)	6.0 ± 3.1	4.8 ± 0.5	0.79 ± 0.23	700 ± 130	25 ± 12	61 ± 30	12.5 ± 5.6
Broadleaf forest (*n* = 25)	5.8 ± 2.4	4.9 ± 0.4	0.80 ± 0.15	860 ± 190	23 ± 11	57 ± 28	10.3 ± 5.4
Bamboo forest (*n* = 21)	4.0 ± 2.3	5.3 ± 0.6	0.87 ± 0.27	520 ± 270	34 ± 9	92 ± 29	17.0 ± 4.0
Shrubland (*n* = 19)	7.1 ± 3.3	5.0 ± 0.6	0.66 ± 0.17	600 ± 270	23 ± 11	61 ± 30	10.8 ± 5.4
Grassland (*n* = 8)	4.2 ± 1.6	4.8 ± 0.4	0.81 ± 0.14	540 ± 290	32 ± 14	82 ± 16	14.6 ± 5.2
Overall (*n* = 150)	5.7 ± 2.8	4.9 ± 0.4	0.80 ± 0.24	620 ± 280	26 ± 12	66 ± 31	12.5 ± 5.5

**Note:**

Values are presented as mean ± standard deviation (SD). SOC, soil organic carbon; BD, bulk density; AG, α-glucosidase; BG, β-glucosidase; CBH, cellobiohydrolase.

### Model comparison

Nested cross-validation showed broadly comparable predictive performance among the three ensemble algorithms (Table 2). Under repeated five-fold cross-validation, RF, BRT, and XGBoost produced nearly identical R^2^ and RMSE values, although XGBoost yielded a marginally lower MAE. Under NNDM-CV, RF showed a slight numerical advantage, but the differences among the three algorithms remained small. Overall, the small differences among RF, BRT, and XGBoost suggest that SOCD prediction was relatively robust across ensemble algorithms. Given this comparable performance, we selected XGBoost for subsequent wall-to-wall SOCD mapping because its built-in regularization suits the limited sample size (*n* = 150) and its gradient-boosted-tree structure integrates directly with TreeSHAP, facilitating the SHAP-based analysis of SOCD drivers.

**Table 2:** Comparison of model performance for soil organic carbon density (SOCD) prediction.

Model	Repeated nested five-fold CV			Nested NNDM-CV		
	R^2^_NSE	RMSE (t C hm^−2^)	MAE (t C hm^−2^)	R^2^_NSE	RMSE (t C hm^−2^)	MAE (t C hm^−2^)
RF	0.59 ± 0.02	22.5 ± 0.5	18.9 ± 0.5	0.58	22.6	18.8
BRT	0.59 ± 0.02	22.5 ± 0.5	18.8 ± 0.4	0.58	22.7	19.2
XGBoost	0.59 ± 0.03	22.5 ± 0.8	18.6 ± 0.7	0.57	22.9	19.2

**Note:**

R^2^_NSE denotes the Nash-Sutcliffe efficiency form of R^2^. RMSE and MAE are expressed in t C hm^−2^. Values for repeated nested five-fold cross-validation are means ± standard deviations across five outer repeats. Nested NNDM-CV values were calculated from the pooled predictions for the 150 outer test observations. Hyperparameters were selected by five-fold inner cross-validation independently within each outer-training partition and therefore could differ among outer folds.

### SOCD prediction and uncertainty

**Figure 3:**
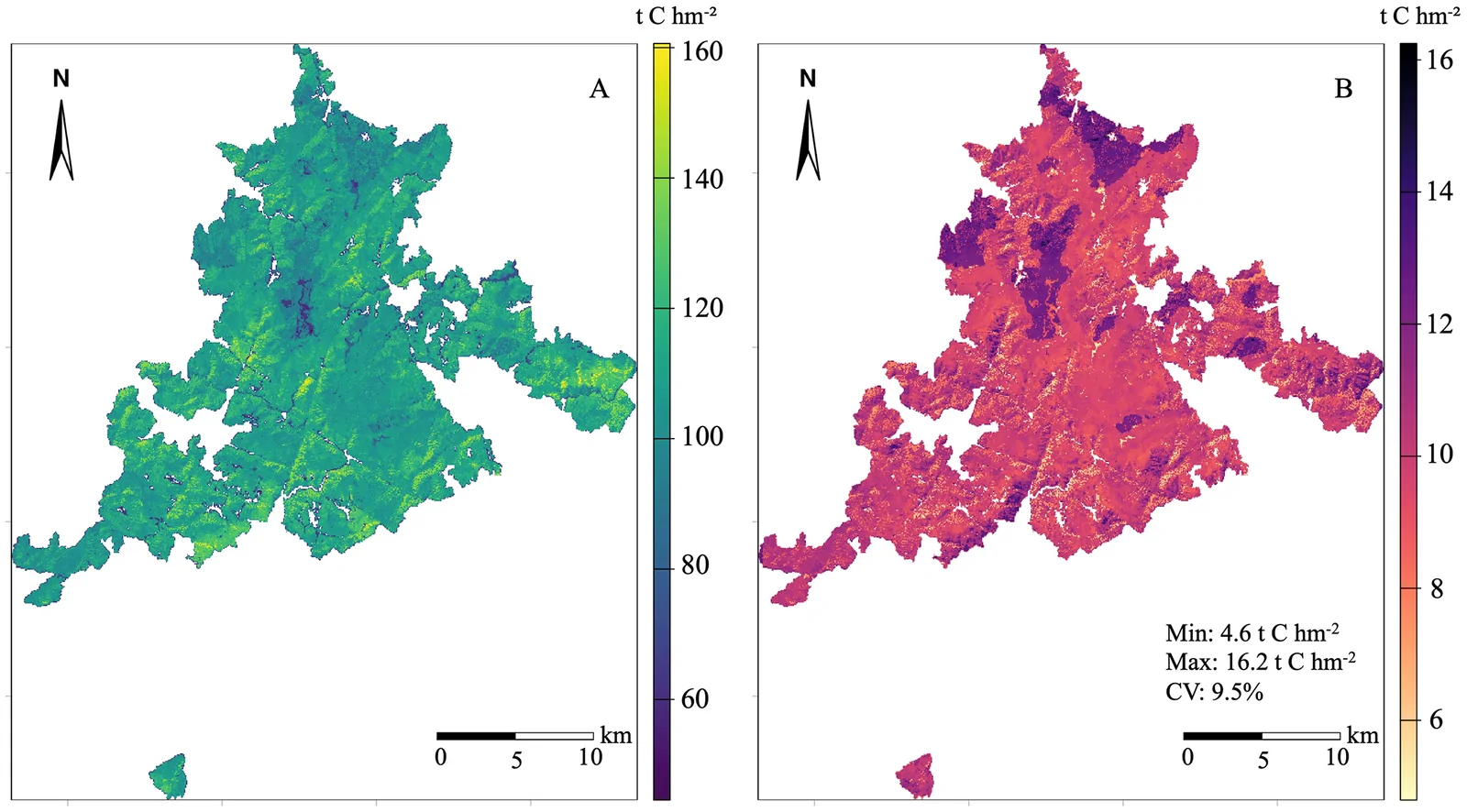
Spatial prediction of soil organic carbon density (SOCD) and associated uncertainty across Baishanzu National Park. (A) Predicted SOCD generated using the final XGBoost model; (B) pixel-level prediction uncertainty arising from bootstrap resampling of the training data, expressed as the standard deviation (SD) of predictions from a 100-member bootstrap XGBoost ensemble.

The predicted surface SOCD across Baishanzu National Park averaged 108.6 t C hm^−2^, with pixel-level values ranging from 39.4 to 160.6 t C hm^−2^ (Fig. 3A). The bootstrap ensemble indicated moderate spatial variation in resampling-based model uncertainty (Fig. 3B). Pixel-level uncertainty, expressed as the standard deviation of the 100 bootstrap XGBoost predictions, averaged 10.0 t C hm^−2^ and ranged from 4.6 to 16.2 t C hm^−2^, with a mean coefficient of variation of 9.5%. We note that these values are smaller than the cross-validated RMSE (22.5 t C hm^−2^), confirming that the bootstrap ensemble reflects only the model component of uncertainty rather than the total prediction uncertainty across the study area.

**Figure 4:**
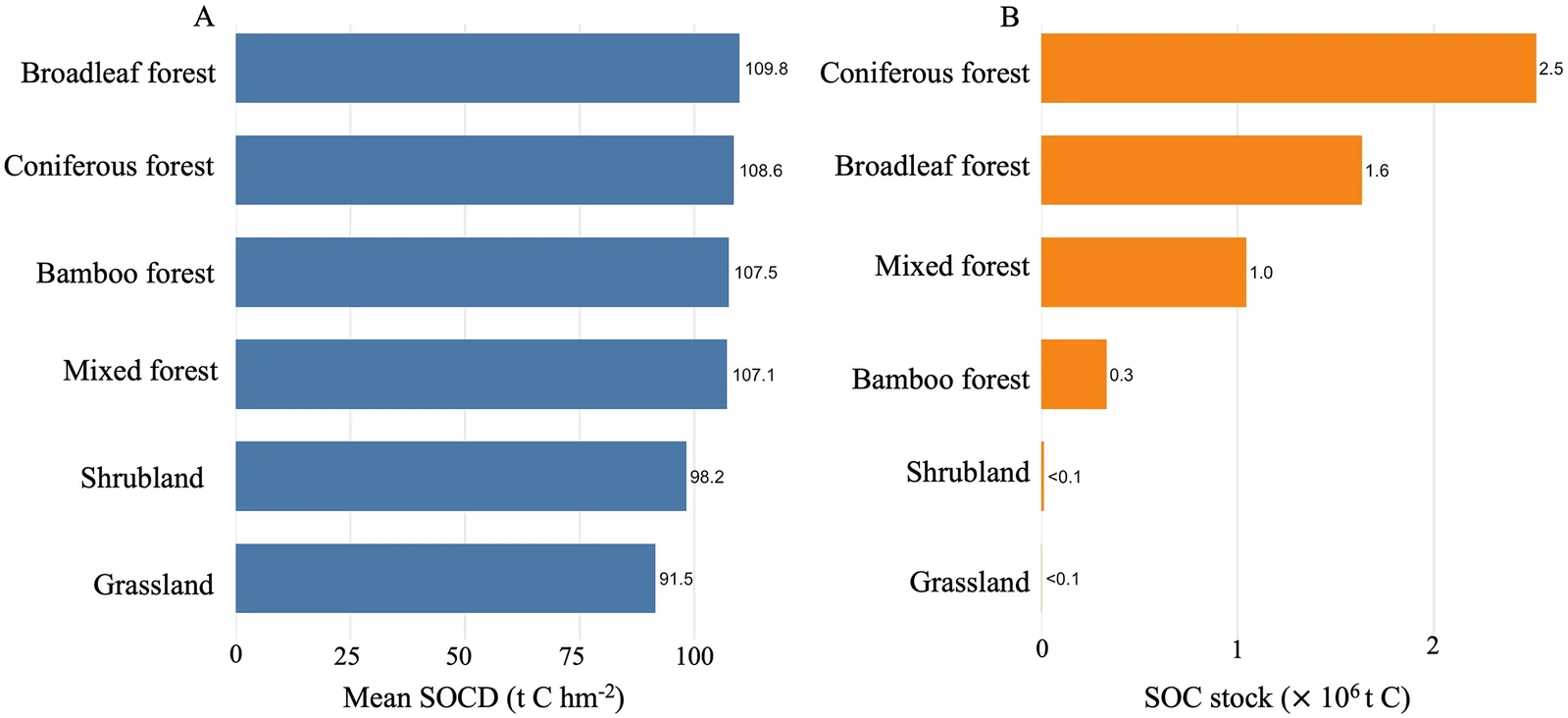
Mean predicted surface SOCD (A) and total surface SOC stock (B) across vegetation types in Baishanzu National Park. SOCD, soil organic carbon density; SOC, soil organic carbon.

Mean predicted SOCD varied among vegetation types, although differences among the dominant forest types were small. Broadleaf forest, coniferous forest, bamboo forest, and mixed coniferous–broadleaf forest showed higher SOCD than shrubland and grassland (Fig. 4A). Based on the raster prediction, the total surface SOC stock within the study area was approximately 5.4 × 10^6^ t C. Because coniferous forest, broadleaf forest, and mixed coniferous-broadleaf forest occupied most of the mapped area, these three vegetation types together accounted for 93.7% of the total predicted SOC stock (Fig. 4B).

### Influencing factors of SOCD distribution

**Figure 5:**
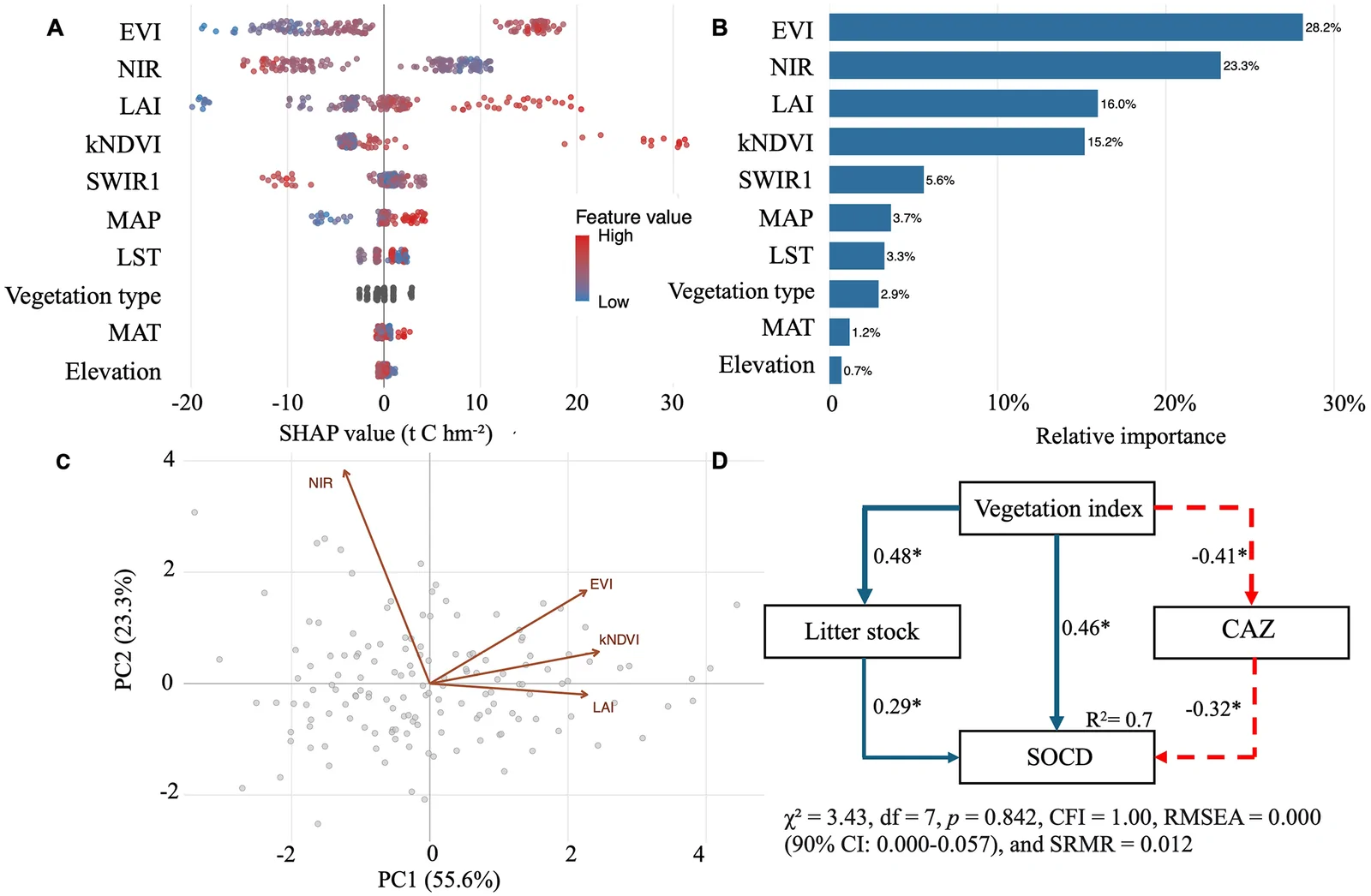
Model interpretation and pathway analysis of factors associated with surface SOCD distribution. (A) SHapley Additive exPlanations (SHAP) summary plot showing the direction and magnitude of each predictor’s contribution to the XGBoost prediction of SOCD. (B) Relative importance of predictors calculated from mean absolute SHAP values. (C) Principal component analysis (PCA) biplot of the four dominant vegetation-related predictors identified by SHAP analysis: EVI, NIR, LAI, and kNDVI. (D) Structural equation model showing direct and indirect associations among the vegetation-index composite, litter stock, C-acquiring enzyme index (CAZ), and SOCD. Blue solid arrows indicate positive paths, and red dashed arrows indicate negative paths. Numbers beside arrows are standardized path coefficients; asterisks indicate significant paths (*p* < 0.05). SOCD, soil organic carbon density; EVI, enhanced vegetation index; NIR, near-infrared; LAI, leaf area index; kNDVI, kernel normalized difference vegetation index; SWIR1, shortwave infrared 1; MAP, mean annual precipitation; MAT, mean annual temperature; LST, land surface temperature.

The spatial distribution of SOCD was mainly explained by vegetation-related variables (Fig. 5). SHAP analysis showed that EVI was the most important predictor, contributing 28.2% of the total relative importance, followed by NIR (23.3%), LAI (16.0%), and kNDVI (15.2%) (Figs. 5A, 5B). PCA of the top-four variables showed that PC1 explained 55.6% of the total variance and was positively associated with EVI, LAI, and kNDVI, whereas PC2 explained 23.3% of the variance and was mainly associated with NIR (Fig. 5C). Thus, PC1 represented a general vegetation greenness and canopy-development gradient.

The SEM further supported the dominant role of the vegetation-index composite represented by PC1 in influencing SOCD (Fig. 5D). The vegetation index had a significant positive direct effect on SOCD (path coefficient = 0.46), and also had an indirect positive effect through litter stock, as it positively affected litter stock (0.48), which in turn increased SOCD (0.29). In contrast, the vegetation index had a negative effect on the C-acquiring enzyme index (−0.41), and the C-acquiring enzyme index was negatively associated with SOCD (−0.32). Overall, the SEM explained 70% of the variation in SOCD, suggesting that vegetation-related variables were associated with SOCD through both direct and indirect pathways consistent with litter accumulation and C-acquiring enzyme activity.

## Discussion

The three ensemble algorithms achieved broadly comparable predictive performance in this topographically complex, vegetation-diverse environment (Table 2). Under repeated nested five-fold cross-validation, RF, BRT, and XGBoost produced nearly identical R^2^ and RMSE, and under nested NNDM-CV, which accounts for the spatial clustering of the sampling design, their differences likewise remained small, indicating that predictive performance was robust both across algorithms and to spatial clustering. We nonetheless selected XGBoost for mapping (Fig. 3A), because its built-in regularization reduces the risk of overfitting when the sample size is limited (*n* = 150) and its gradient-boosted-tree structure integrates directly with TreeSHAP (Chen & Guestrin, 2016). Using a 100-member bootstrap XGBoost ensemble, we obtained a spatially explicit estimate of pixel-level resampling and model-fitting uncertainty (Fig. 3B). This uncertainty was moderate relative to the predicted SOCD (mean coefficient of variation ≈ 9.5%), indicating that the broad spatial pattern of the map was comparatively stable across bootstrap resamples. It should be noted that this bootstrap ensemble captures uncertainty arising from sampling variability and model fitting, but not uncertainty in the input covariates or the change of support involved in aggregating pixel predictions to a regional total; rigorous quantification of whole-park (areal) uncertainty would require geostatistical simulation (Zhu et al., 2025). The reported total surface SOC stock should therefore be regarded as a best estimate rather than an uncertainty-bounded value.

The mean surface SOCD of Baishanzu National Park (108.6 t C hm^−2^) is higher than the values reported for several comparable protected areas in China, such as Wuyishan National Park (~83.5 t C hm^−2^) (Chen et al., 2023), the Giant Panda National Park (~68.5 t C hm^−2^) (Ma, 2020), and Maoer Mountain National Forest Park (~62.5 t C hm^−2^) (Jiang, Gao & Cui, 2015). This high SOCD may reflect the convergence of several favorable conditions. First, Baishanzu’s vegetation is dominated by mature coniferous and evergreen broadleaf forests, which produce high and sustained litter inputs and sustain dense root networks (Zhang et al., 2023). Second, the mid-subtropical montane climate combines high annual precipitation (>2,300 mm) with moderate temperatures, maintaining consistently high soil moisture that slows aerobic decomposition and promotes organic matter preservation (Hao et al., 2025). Third, the limited degree of anthropogenic disturbance in the conservation zone has allowed the progressive accumulation of SOC over decades without the losses associated with land-use change, logging, or tourism trampling.

Mean predicted SOCD was higher in the dominant forest types, including broadleaf, coniferous, and mixed coniferous–broadleaf forest, than in shrubland and grassland (Fig. 4A). The three dominant forest types together accounted for 93.7% of the total predicted surface SOC stock (Fig. 4B). SHAP analysis of the XGBoost model identified vegetation-related indices as the dominant predictors of surface SOCD distribution (Figs. 5A, 5B). This is consistent with previous literature showing that vegetation productivity is the principal driver of SOC spatial heterogeneity in forest landscapes (Vitharana et al., 2024). SHAP, however, quantifies each covariate’s contribution to the prediction without distinguishing whether that contribution is direct or mediated by other, correlated processes. To complement this predictive interpretation and evaluate hypothesized ecological pathways, we therefore fitted an SEM to the field-measured process variables (Fig. 5D). The SEM identified three distinct pathways through which vegetation influences SOCD and explained 70% of the variation in SOCD overall. Vegetation had a direct positive effect on SOCD (β = +0.46). This may suggest that root C inputs can deliver C directly to the mineral soil independently of surface litter dynamics (Sokol et al., 2019). The positive indirect pathway through litter stock was consistent with the interpretation that higher vegetation productivity may be associated with greater surface C inputs (Khulpu et al., 2025). Notably, the vegetation index also exerted a negative indirect effect on SOCD *via* the C-acquiring enzyme index (Fig. 5D). This pattern suggests that high-productivity vegetation is associated with lower cellulolytic hydrolase activity, which may weaken the microbial decomposition of SOC. This interpretation is broadly consistent with Luo et al. (2025), who found that high plant productivity could reduce microbial C limitation and therefore decrease the C-acquiring enzyme activities. We emphasize, however, that these are statistically inferred relationships consistent with the hypothesized pathways rather than demonstrated causal mechanisms; alternative explanations—including substrate availability, microbial community adaptation, and confounding soil properties (*e.g*., texture, moisture, or pH)—cannot be excluded.

Several limitations should be considered. First, our analysis was restricted to the 0–20 cm topsoil; although this layer is the most biologically active and holds a large share of profile SOC, the reported densities and stock represent surface SOC only and should not be equated with whole-profile or whole-ecosystem C. Second, sampling was based on a single campaign (July–November 2024); whereas SOC changes slowly, enzyme activities and litter inputs can vary seasonally, which may affect the SEM relationships. Future work incorporating deeper soil layers, repeated seasonal sampling, and additional edaphic covariates would help to test and generalize these findings.

## Conclusions

This study combined 150 topsoil (0–20 cm) sampling sites with machine-learning algorithms to predict and map surface SOCD across Baishanzu National Park, and used SHAP to interpret the prediction model and a separate SEM to evaluate hypothesized ecological pathways among field-measured variables. The main findings were: (1) the predicted mean surface SOCD was 108.6 t C hm^−2^, corresponding to a total surface SOC stock of approximately 5.4 × 10^6^ t C; (2) vegetation-related spectral indices (EVI, NIR, LAI, and kNDVI) were the dominant predictors of SOCD spatial variation; (3) SOCD was higher in broadleaf, coniferous, and mixed forests than in shrubland and grassland, with the three dominant forest types accounting for 93.7% of the total surface SOC stock; and (4) SEM indicated that vegetation productivity is associated with SOCD both directly and indirectly, explaining 70% of SOCD variation. Overall, the results clarify the spatial distribution and proximate drivers of surface SOC in subtropical montane forests and provide a basis for regional surface-C assessment and forest management.

## Supplemental Information

10.7717/peerj.21704/supp-1 Supplemental Information 1 Code for fitting, tuning, and evaluating the Random Forest, Boosted Regression Trees, and XGBoost models.

10.7717/peerj.21704/supp-2 Supplemental Information 2 Nested validation.

10.7717/peerj.21704/supp-3 Supplemental Information 3 Raw data of soil organic C and associated properties.

## Additional Information and Declarations

### Field Study Permissions

The following information was supplied relating to field study approvals (*i.e*., approving body and any reference numbers):

No separate field permit number or external approval document was required for this study. Fieldwork was internally authorized by Qianjiangyuan-Baishanzu National Park Qingyuan Conservation Center.

### Data Availability

The following information was supplied regarding data availability:

The data and code are available in the Supplemental Files.
